# Description of a Novel Mycovirus in the Phytopathogen *Fusarium culmorum* and a Related EVE in the Yeast *Lipomyces starkeyi*

**DOI:** 10.3390/v12050523

**Published:** 2020-05-09

**Authors:** Mathieu Mahillon, Gustavo Romay, Charlotte Liénard, Anne Legrève, Claude Bragard

**Affiliations:** UCLouvain, Earth and Life Institute, Applied Microbiology-Phytopathology, Croix du Sud 2-L07.05.03, 1348 Louvain-la-Neuve, Belgium; mathieu.mahillon@uclouvain.be (M.M.); gromay@gmail.com (G.R.); charlotte.lienard@uclouvain.be (C.L.); anne.legreve@uclouvain.be (A.L.)

**Keywords:** mycovirus, dsRNA, Unirnaviridae, *Fusarium culmorum*, EVE, *Lipomyces starkeyi*

## Abstract

A new mycovirus was found in the *Fusarium culmorum* strain A104-1 originally sampled on wheat in Belgium. This novel virus, for which the name Fusarium culmorum virus 1 (FcV1) is suggested, is phylogenetically related to members of the previously proposed family ‘’Unirnaviridae’’. FcV1 has a monopartite dsRNA genome of 2898 bp that harbors two large non-overlapping ORFs. A typical -1 slippery motif is found at the end of ORF1, advocating that ORF2 is translated by programmed ribosomal frameshifting. While ORF2 exhibits a conserved replicase domain, ORF1 encodes for an undetermined protein. Interestingly, a hypothetically transcribed gene similar to unirnaviruses ORF1 was found in the genome of *Lipomyces starkeyi*, presumably resulting from a viral endogenization in this yeast. Conidial isolation and chemical treatment were unsuccessful to obtain a virus-free isogenic line of the fungal host, highlighting a high retention rate for FcV1 but hindering its biological characterization. In parallel, attempt to horizontally transfer FcV1 to another strain of *F. culmorum* by dual culture failed. Eventually, a screening of other strains of the same fungal species suggests the presence of FcV1 in two other strains from Europe.

## 1. Introduction

Mycoviruses have been described in all major fungal taxa and more extensively in genera with economic or medical importance [1]. Amongst those, the genus *Fusarium* has received much attention leading to the discovery of many mycoviruses, especially in *Fusarium oxysporum* and *Fusarium graminearum* [2,3]. In this study, we characterize for the first time a virus from the phytopathogenic species *F. culmorum*.

*F. culmorum* belongs to the fungal complex causing Fusarium head blight and Fusarium foot and root rot on several plant species [4]. In addition to its pathogenicity, *F. culmorum* is known to produce mycotoxins that contaminate crop products, hampering their consumption by humans and cattle [5,6]. Currently, the control of this pathogen relies on the extensive use of fungicides. However, besides the potentially detrimental effect of such chemicals on the environment, *F. culmorum* has developed related resistance [7]. Thus, there is a need for alternative control strategies. Mycoviruses conferring hypovirulence have been proposed for this purpose [8], motivating the search for such potential biocontrol agents.

The majority of known mycoviruses are made of dsRNA [1], hence screening for dsRNA in fungal mycelia is a fast method to discover new mycoviruses. Despite the fact that dsRNA molecules have been previously detected in *F. culmorum*, no viral sequence has been described yet [9,10]. By screening our laboratory collection of this fungal species, we found a dsRNA element in the strain A104-1. This dsRNA was fully sequenced and was putatively assigned to a new viral species for which primary bioinformatics and biological analyses are given.

## 2. Materials and Methods

### 2.1. Recovery of FcV1 dsRNA

*Fusarium culmorum* virus 1 (FcV1) was recovered from the *F. culmorum* strain A104-1. This strain was originally sampled in Corroy-le-Grand (Belgium) on wheat in 2008. Identification and handling of the fungus was done as previously described [11]. DsRNA extraction was done on 100 mg of dried mycelium according to a cellulose-based protocol [12]. The dsRNA nature of the detected band was proven by resistance to treatment with DNase 1 and S1 nuclease (Thermo Scientific, Waltham, MA, USA). Following agarose gel electrophoresis, the gel band containing the dsRNA was excised and incubated overnight (O/N) in 100 µL of milliQ water at 4 °C. After centrifugation at 1000× *g* for 1 min, the supernatant was collected and subjected to ethanol precipitation. The obtained pellet was resuspended in a final volume of 15 µL of milliQ water.

### 2.2. Full Genome Sequencing

A volume of 5 µL of the purified dsRNA solution was used to perform a reverse transcription using the M-MLV reverse transcriptase (Promega Madison, WI, USA) with the degenerate primer 5′-GACGTCCAGATCGCGAATTCNNNNNN-3′, according to Wang et al. [13]. The resulting cDNA was amplified by PCR using the GoTaq polymerase (Promega Madison, WI, USA) and including the primer 5′-GACGTCCAGATCGCGAATTC-3′. Two major amplicons were obtained and cloned inside the pGEM-T Easy vector (Promega Madison, WI, USA). The plasmids were then used to transform chemically competent *E. coli* 10β cells. The plasmids were subsequently sequenced using the forward and reverse M13 universal primers. Then, primers corresponding to the viral sequences were designed and used to obtain the sequence of the gap between the obtained amplicons.

The 5′- and 3′-termini were sequenced according to Xie et al. [14]. Briefly, the 5′-phosphorylated oligonucleotide 5′-GCATTGCATCATGATCGATCGAATTCTTTAGTGAGGGTTAATTGCC-NH_2_-3′ was ligated on both extremities of the dsRNA using T4 RNA ligase (Thermo Scientific, Waltham, MA, USA), O/N at 4 °C. Reverse transcription was then performed using the complementary primer 5′-GGCAATTAACCCTCACTAAAG-3′, with the same enzyme already mentioned. Subsequently, the cDNA was amplified using the primer 5′-TCACTAAAGAATTCGATCGATC-3′ in combination with the viral specific primers 5′-TCCGAAAGGATGCTCG-3′ or 5′-GAATGAGTTTGGAATGAACATT-3′. Amplicons were directly sequenced.

### 2.3. Genome Sequence Analysis

The whole genome of FcV1 was assembled using UGene [15]. The RNA secondary structure of FcV1 3′-termini was predicted via the Mfold server, using default parameters except the temperature set at 25 °C [16]. For analysis of ORF1p of unirnaviruses, ustiviruses and amalgaviruses, sequences were retrieved from Genbank database (Table S1). Sequences were aligned via MUSCLE [17]. A pairwise identity matrix was obtained via the SDT software [18]. A similarity plot for the alignment of unirnaviruses ORF1p was generated using Plotcon (http://www.bioinformatics.nl/cgi-bin/emboss/plotcon, with default parameters) and the prediction of coiled coil was made using Deepcoil [19]. Remote homologs were searched using the HMMERsearch software [20] with the aligned sequences of unirnaviruses ORF1p as a query. For analysis of ORF2, sequences were first retrieved from the Genbank database (Table S2). Then, sequences were aligned via MUSCLE. For phylogenetic analysis, the best fit substitution model (rtREV+F+I+G4) for this alignment was found via ModelFinder [21] and used to create a maximum-likelihood phylogenetic tree via IQ-TREE [22], with ultrafast bootstrap (1000 replicates) [23]. The tree was visualized using MEGA-X [24]. Alternatively, a phylogenetic tree was also obtained using MrBayes (with rtREV model) [25] and visualized using MEGA-X.

### 2.4. Evaluation of Vertical Transmission

The *F. culmorum* strain A104-1 was grown on synthetic nutrient-poor agar (SNA) medium for 10 days at room temperature (RT). Macroconidia were harvested by adding and collecting 1 mL of sterile water containing Tween-20 (one drop per mL) to the cultures. The suspension was then poured on 3% (*w*/*v*) agar plates with serial dilutions and incubated O/N at RT. Sixty germinated macrononidia were individually transferred onto potato dextrose agar (PDA) plates and subsequently incubated for 10 days at RT. Total RNA was extracted from the produced mycelia using Trizol reagent (Invitrogen, Waltham, MA, USA) according to the manufacturer’s instructions. Then, RNA was subjected to RT-PCR using the specific primers 5′-GGCGTTAAGGCGTTGGGCTACT-3′ (positions 1701–1722) and 5′-CCTCGACCCGCAACGCC-3′ (positions 2200–2184) with the enzymes already described.

### 2.5. Chemical Treatment Assay

Cycloheximide and rifampicin were used in the aim to obtain a virus-free isogenic line of the fungus. Briefly, 20 mL of carboxymethyl cellulose (CMC) liquid medium supplemented with either cycloheximide (1 mg/L) or rifampicin (1 mg/L) were inoculated with plugs of mycelia and incubated at 120 rpm at 25 °C in darkness. After 5 days of culture, conidia were collected and poured onto PDA supplemented with the same chemicals. Fourteen individual fungal colonies were isolated for each treatment and sub-cultured for at least three serial passages via hyphal-tipping on similar media. Afterwards, mycelia were grown on PDA without chemical and screened for the presence of FcV1 by RT-PCR as already described.

### 2.6. Horizontal Transmission Assay

A mycelia plug of the fungal strain A104-1 was grown on PDA next to a mycelia plug from the *F. culmorum* strain P1P2 that was obtained from the strain UK99 after repeated passages on tebuconazole supplemented media [7]. Seven days after co-culture, 6 mycelia plugs located on the confrontation zone were transferred onto PDA containing tebuconazole (1 mg/L) for selection. Plugs from fast growing mycelia sectors were then transferred onto PDA and screened for the presence of FcV1 by RT-PCR as already described.

### 2.7. Prevalence of FcV1 in Other F. culmorum Strains

Fifty-three strains of F. culmorum originated from diverse locations (Table S3) were grown on PDA for 10 days at RT. Following this, the produced mycelia were screened for the presence of FcV1 by RT-PCR, as described above. Strains that were positive were further analysed by dsRNA analysis.

## 3. Results

### 3.1. Genome Sequence Analysis of FcV1

**Figure 1:**
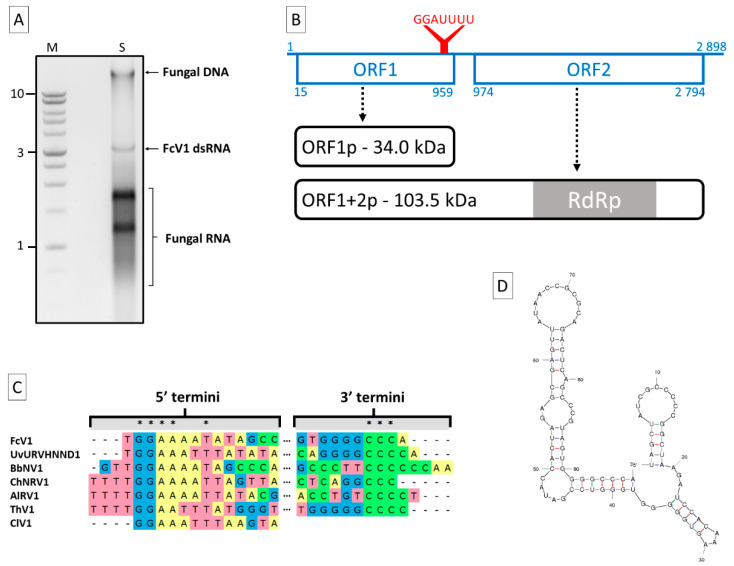
*Fusarium culmorum* virus 1 (FcV1) genome characterization. (**a**) Electrophoresis agarose (1%) gel showing fungal DNA, FcV1 dsRNA and fungal RNA extracted from *F. culmorum* A104-1. M: DNA weight markers (Promega 1 kb ladder). S: extracted sample. Numbers on the left refer to chosen DNA molecular weights expressed in kilobase; (**b**) schematic representation of FcV1 monopartite genome (blue lines). The straight line represents the genomic (+) strand RNA. Boxes represent ORF with associated positions. The slippery motif is highlighted via the red Y. Hypothetical translation products (black curved boxes) are indicated by dotted arrows; (**c**) alignment of termini of FcV1 cDNA with other selected unirnaviruses. Asterix highlight conserved residues; (**d**) predicted stem-loop structure for FcV1 3′-UTR.

Following a dsRNA extraction on the *F. culmorum* strain A104-1, one major band of ca. 3 kb was visible on agarose gel in addition to fungal DNA and RNA (Figure 1A). The dsRNA nature of this band was proven by resistance to DNase I and S1 nuclease (Figure S1). An RT-PCR on this dsRNA using a degenerate primer yielded two amplicons (Figure S2) that allowed to start the sequencing. After completing the latter, the full sequence was determined to be 2898 bp in length. Due to its dsRNA nature, the sequence was assigned to the genome of a novel virus for which the name *Fusarium culmorum* virus 1 (FcV1) is proposed. The full sequence has been deposited in the Genbank database under the accession number MN187541. A BLASTn search for the total nucleotide sequence resulted in significant hits (E-value ≤ 4 × 10^−10^, 5–80% coverage) corresponding to members of the proposed family “Unirnaviridae”. Like other members of this group (see Table 1 for viruses abbreviations and characteristics), FcV1 genome harbors two large non-overlapping ORF (ORF1 and 2) on the positive strand, at positions 15–959 and 974–2794, respectively (Figure 1B). ORF1 encodes for a putative protein of 314 aa with an estimated mass of 34.02 kDa. The typical -1 slippery motif G_GAU_UUU [26] is found at the 3′ end of ORF1 (positions 950–956), suggesting that ORF2 is translated by programmed ribosomal frameshifting (PRF). This PRF hypothesis is supported for unirnaviruses by the fact that a protein with homology to both ORF1 and ORF2 was recovered during an investigation on ChNRV1 translation products [27]. In the case of FcV1, the hypothetically produced protein ORF1+2p contains 926 aa, with an estimated mass of 103.53 kDa.

The 5′- and 3′-UTR of FcV1 genome are 14 and 104 bp long, respectively. An alignment of unirnaviruses 5′-termini evidenced the conserved motif GGAAA/UA/UUA/U in FcV1, UvURVHNND1, BbNV1, ChNRV1, AlRV1, ClV1 and ThV1 (Figure 1C). An alignment of unirnaviruses 3′-termini only highlights the presence of series of C residues of variable lengths (3 to 6) in FcV1, UvURVHNND1, BbNV1, ChNRV1, AlRV1 and ThV1 (Figure 1C). The termini of other unirnaviruses do not contain these motifs, probably due to incomplete sequencing. Interestingly, FcV1 3′-UTR is predicted to fold into stable stem-loop structures (Figure 1D, with minimum optimal energy ΔG= −37.32 kcal/mol). This prediction was also evidenced for several related viruses [28] and such secondary structures might be of biological importance in terms of replication and/or translation, a common feature of viral UTRs.

### 3.2. Analysis of FcV1 ORF1p and Retrieving of ORF1p Homologs

**Figure 2:**
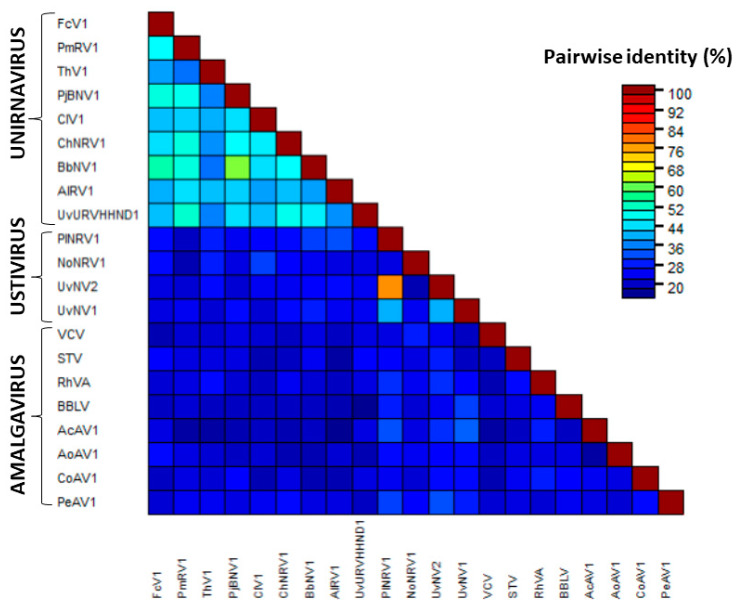
Pairwise identity matrix of ORF1p of unirnaviruses, ustiviruses and several amalgaviruses.

As expected by nucleotide similarities, a BLASTp search for FcV1 ORF1p resulted in significant hits (E-value ≤ 5 × 10^−50^, ≥ 69% coverage) corresponding to the predicted ORF1p of other unirnaviruses. All those proteins are similar in size (314–394 aa, except PmRV1 for which ORF1p might be not fully sequenced given the absence of the conserved 5′-terminus) and show 34–61% pairwise identity (Figure 2).

**Figure 3:**
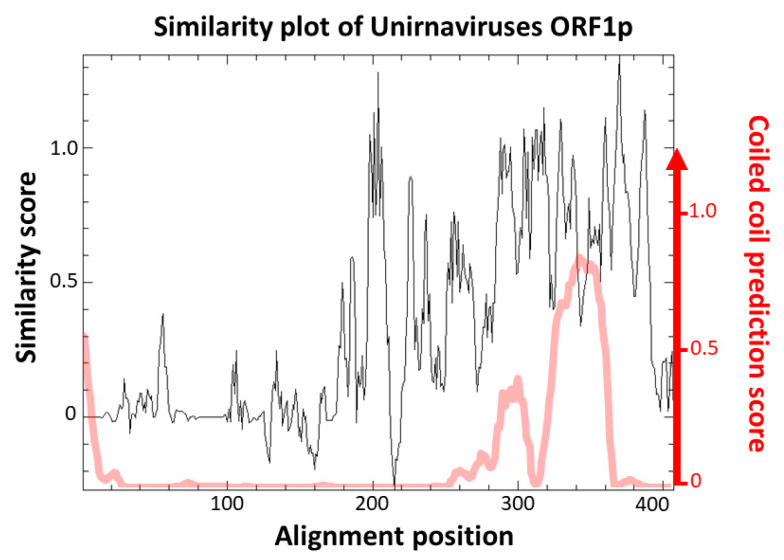
Similarity plot of aligned sequences of unirnaviruses ORF1p (black line) and CC prediction using Deepcoil (red line).

The function of those proteins is currently unknown. Campo et al. [27] pointed out a high similarity score between the predicted topology of ChNRV1 ORF1p and a portion of the capsid protein (CP) of Saccharomyces cerevisiae virus L-A. However, no true viral particle has been associated with any unirnavirus by transmission electron microscopy. Instead, it was shown that BbNV1 most likely exhibit linear chain-like structures when analyzed by atomic force microscopy [36]. Those are believed to be a particular association of the genomic dsRNA with ORF1p [36]. The absence of true virion is also observed for two other related groups of monopartite dsRNA viruses, namely ustiviruses and amalgaviruses (see Section 3.3), which also have a ca. 3 kb genome encoding for two ORFs and containing a PRF motif. While ORF1p of ustiviruses are much smaller (ca. 180 aa) than those of unirnaviruses, those of amalgaviruses are more similar in size (ca. 380 aa). ORF1p from these three viral groups show small pairwise identity (Figure 2), yet Nibert et al. [37] have shown that they all exhibit an α-helical coiled coil (CC) in their predicted tertiary structures. A similarity plot of an alignment of FcV1 and other unirnavirus ORF1p (Figure 3, black line) shows that the N-terminal region is highly variable while the C-terminal region containing the predicted CC (Figure 3, red line) is conserved, suggesting the importance of this structure in unirnaviruses ORF1p function.

Interestingly, it was proposed that amalgaviruses ORF1p originated from a nucleocapsid protein (NC) from a phlebo/tenuivirus during a genomic recombination event, involving a partitivirus as the donor for the replicase [38]. This hypothesis was supported by results obtained from searches using softwares based on pairwise comparison of profile hidden Markov models (HMM). When similar searches were carried out for unirnaviruses ORF1p, no proteins corresponding to known phlebo/tenuivirus NC were listed in the hits. Instead, using an alignment of unirnaviruses ORF1p as a query for a search using HMMERsearch, three significant hits were obtained corresponding to uncharacterized proteins: A0A2V0RJY0, A0A2V0RJC8 and A0A2V0RBA2 (E-value ≤ 5.2 × 10^−9^). Those proteins are longer (544, 488 and 546 aa, respectively) than unirnaviruses ORF1p but contain a similar CC in their respective C-terminal region (Figure S3). They were predicted from a metagenomic study in marine microorganisms in the north-west Pacific Ocean [39]. No other information is available regarding those sequences besides the fact that their assembling reads were obtained following a dsRNA extraction protocol from cells collected from surface water, suggesting than they represent sequences of dsRNA viruses infecting those organisms.

Unexpectedly, a hypothetical protein (ODQ71866.1) from *Lipomyces starkeyi* strain NRRL Y-11557 [40] was also evidenced by a BLASTp search for FcV1 ORF1p (E-value = 2 × 10^−16^, 55% coverage). A total of 153 out of 187 aa (82%) of this protein shows strong similarity to the conserved C-terminal region of unirnaviruses ORF1p, although its total length is much shorter. Such a result could correspond to an endogenous virus-like element (EVE), resulting from the integration of a viral cDNA into its host genome.

**Figure 4:**
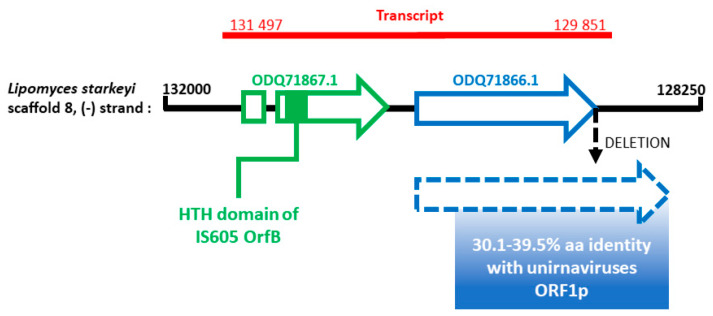
Schematic representation of the portion of *L. starkeyi* genome (black horizontal line) harboring the gene homolog to unirnaviruses ORF1p (blue arrow) as well as the co-transcribed gene harboring the helix-turn-helix (HTH) domain (green arrow). The putative transcript is shown (red line). The positions are given respectively to the (+) strand.

To get more insights on this potential EVE, we used the MycoCosm portal [41]. We retrieved the gene of interest inside the *L. starkeyi* genome in the scaffold 8 at positions 129932–130495, on the minus strand (Figure 4, blue arrow). This gene seems to be unique to *L. starkeyi* since a BLASTp search did not reveal any significant hit in other eukaryotic genomes, advocating for an integration event. A nucleotide deletion in the stop codon of the ORF prolongs the coding region by 192 nt, leading to an additional fragment of 64 aa (Figure 4, dotted blue arrow). Remarkably, this additional fragment also displays similarity with unirnaviruses ORF1p. Overall, the pairwise aa identity of this region ranges from 30.1 to 39.5% with homolog regions of unirnaviruses ORF1p. According to expressed sequence tag (EST) data, it seems that this gene is transcribed (Figure 4, red line). Interestingly, another gene is found on the same transcript (ODQ71867.1; Figure 4, green arrow) This gene contains an intron and produces a hypothetical protein exhibiting a conserved helix-turn-helix (HTH) domain homolog to that of OrfB of IS605 transposase (pfam:12323, E-value = 10^-12^). However, the remaining sequence does not match to any other characterized protein. Nevertheless, it is tempting to speculate that this protein might have been involved in the horizontal gene transfer event that led to the viral endogenization.

### 3.3. Analysis of FcV1 ORF2 and Phylogenetic Analysis

**Figure 5:**
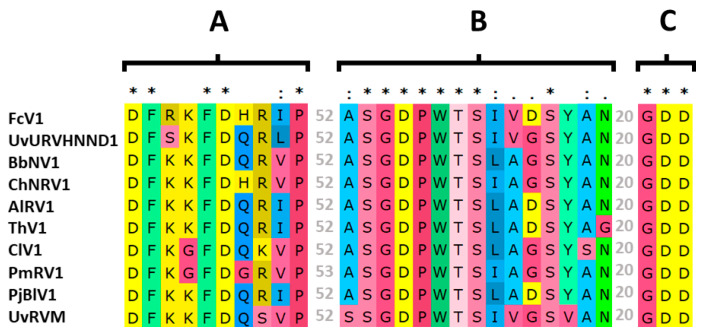
Alignment of unirnaviruses motifs A, B and C from the RNA-dependent RNA-polymerase (RdRp) palm domain. Identical residues are highlighted by asterisks, highly conserved residues by double dots and less conserved but related residues by single dots. Numbers of residues separating the different motifs are given in grey.

As mentioned above, FcV1 ORF2 is probably translated by a -1 PRF to produce the fusion protein ORF1+2p. While the function of FcV1 ORF1p in the viral cycle remains elusive, FcV1 ORF1+2p is likely to be associated with viral replication. Indeed, ORF2 sequence contains a predicted RNA-dependent RNA-polymerase (RdRp) domain (cd01699, E-value = 2.84 × 10^−16^) at positions 239–433. Inside this RdRp domain, the palm motifs A, B and C [42] were readily identified in FcV1 as well as in other unirnaviruses (Figure 5).

**Figure 6:**
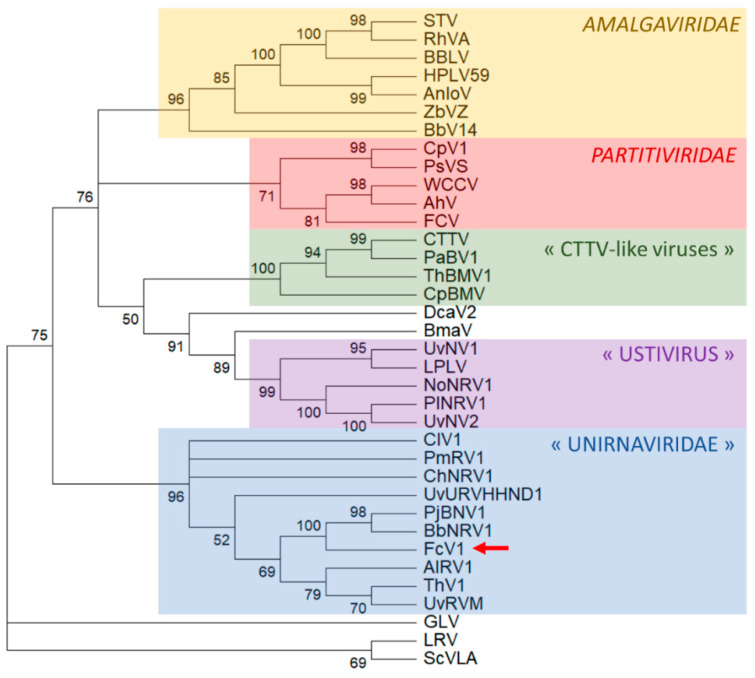
Phylogenetic placement of FcV1 ORF2 and related RdRp. The numbers next to nodes refer the percentage of bootstrap replicates that accommodates the branches together. Branches with bootstrap values <50% were collapsed. The position of FcV1 is highlighted by a red arrow. Members of the family *Totiviridae*: Giardia lamblia virus (GLV), Leishmania RNA virus (LRV) and Saccharomyces cerevisiae virus L-A (ScVLA) were taken to root the initial tree.

A maximum-likelihood phylogenetic analysis based on an alignment of FcV1 ORF2 with related ORF strongly places FcV1 together with other unirnaviruses (Figure 6). A similar result was obtained following a Bayesian method (Figure S4). The proposed family “Unirnaviridae” is related to several other clusters of monopartite viruses with similar genome architecture belonging to the family *Amalgaviridae* and another group for which the genus name ‘’Ustivirus’’ has been proposed [43]. The family *Amalgaviridae* accommodates the genus Amalgavirus as well as the proposed genera ‘’Zybavirus’’ and ‘’Anlovirus’’ [26,28]. While amalgaviruses infect plants, members of “Zybavirus” and “Anlovirus” have been found in the yeast *Zygosaccharomyces bailii* and in the microsporidian *Anlospora locustae*, respectively [26,28]. As far as ustiviruses are concerned, they have been detected in fungi [43].

Recently, putative new viral species related to amalgaviruses and ustiviruses have been detected in arthropods: Beihai barnacle virus 14 (BbV14), Hubei partiti-like virus 59 (HPLV14) and Leshenault partiti-like virus (LPLV) [44,45]. However, these viral sequences were obtained from deep sequencing of total nucleic acids extracted from arthropods, leaving open the possibility that their actual hosts might be arthropod endosymbionts rather than the arthropods themselves. Two viruses somehow related to ustiviruses have been detected in marine microorganisms, namely Bryopsis mitochondrion-associated virus (BmaV) and Diatom colony-associated dsRNA virus 2 (DcaV2) [44,45]. Notably, those viruses also exhibit a similar genome organization consisting of two ORF and a PRF motif.

Despite exhibiting the genome organization of *Totiviridae*, all the aforementioned viruses are actually more related to members of the family *Partitiviridae* and a group of bipartite viruses which were previously referenced as “CTTV-like viruses”, named after the famous Curvalaria thermal tolerance virus [28]. Both viral groups are associated with isometric particles. While CTTV-like viruses have been found exclusively in fungi, partitiviruses are multipartite viruses infecting fungi, plants, protists and insects.

### 3.4. Biological Characterization and Prevalence in Other Strains

In order to assess the vertical transmission rate of FcV1, sixty macroconidia produced by the *F. culmorum strain* A104-1 were isolated and allowed to produce new mycelium. An RT-PCR screening showed that 100 % of those progeny mycelia were infected with the virus, highlighting the ability of FcV1 to be efficiently vertically transmitted in its host.

Since conidial isolation did not result in the loss of FcV1, the fungal host was treated with two different chemicals known for their ability to cure of mycovirus, namely ribavirin [46] and cycloheximide [47]. An RT-PCR screening of mycelia produced by chemically treated conidia revealed the presence of FcV1 in all of them, suggesting the inability of the two chemicals to cure the virus, at least in the tested experimental conditions.

As an alternative to the production of a virus-free isogenic line of the strain A104-1, another way towards the biological characterization of FcV1 would be the infection of a virus-free strain. A dual culture combining the strain A104-1 and the selectable strain P1P2 was achieved to this end. In practical terms, both strains were cultured on PDA and mycelia plugs from the confrontation zone were subcultured on selective media. None of the produced mycelia gave positive signal for FcV1 by RT-PCR. At this point, it is unclear whether this lack of transmission is due to the inability of FcV1 to be transmitted in this fashion or if viral transfer is impeached by vegetative incompatibility between both fungal strains.

The presence of FcV1 in other *F. culmorum* strains was also investigated. In order to do so, fifty-three strains that were originally sampled in Europe, Russia, Turkey, New Zealand and Canada were tested. Interestingly, two European strains gave the amplicon with the expected size (499 bp) by RT-PCR and exhibited a similar ca. 3 kb band when analyzed for the presence of dsRNA (Figure S5): PVS-Fu 353 from Pozzo S. Nicola (Sassari in Sardinia, Italy) [48] and Fc30 from Vaihingen (Germany) [49].

## 4. Discussion

Kotta-Loizou et al. [30] originally suggested the name “Unirnavirus” when they described BbNV1 that was phylogenetically related to AlRV1, implying the creation of a new group to gather both viruses. Since then, several mycoviruses with related RdRp have been discovered in different fungal species, leading the same authors to propose the viral family name “Unirnaviridae” [36]. In addition to related RdRp, all those viruses share the following features: a monopartite dsRNA genome of ca. 3 kb that exhibits two non-overlapping ORF and a -1 PRF motif. As another common characteristic of unirnaviruses, we highlighted conserved RNA motifs at both termini. FcV1 exhibits all the aforementioned features and therefore represent a new member of this putative family.

In this study, we also reported the detection of a unirnaviral EVE in the genome of the yeast *L. starkeyi*. Interestingly, a similar result was found for ORF1p from the related Ustilaginoidea virens nonsegmented virus 1 (UvNV1) [50]. Indeed, UvNV1 ORF1p shows homology with a putative protein encoded by the yeast *Wickerhamomyces ciferrii*. More generally, many EVE have been found in yeasts [51,52], as well as in other eukaryotic genomes [53]. Sometimes, those EVE exhibit large expressed ORF and are located next to transposable elements. We also observed those features for the unirnaviral EVE. Interestingly, it was recently shown that the yeast *Debaryomyces hansenii* produces a counterfeit viral capsid protein derived from an EVE that interferes with the assembly of a similar virus, acting therefore as an antiviral immunity system [54]. Whether the EVE found in *L. starkeyi* could act in a similar way would be worth testing. But at first, confirming the existence and expression of the detected EVE by (RT)-PCR is a prerequisite. 

We were unable to cure the strain A104-1 from FcV1 by isolation of macroconidia. This vertical transmission rate of 100% was already observed for UvNV1 [50]. In parallel, the amalgavirus *Southern tomato virus* was shown to be seed-transmitted at rates ranging from 70 to 90% [55]. Altogether, it seems that those related viruses have evolved efficient strategies to be vertically retained in their respective host. 

Since we were not able neither to cure the virus from the strain A104-1 nor to infect another strain, the biological effect of FcV1 remains undetermined. Previously, it was shown that ChNRV1 induces conidial defects on its host strain mutated for dcl1, whereas it seems to be asymptomatic in the wild-type strain [27]. As far as the related amalgaviruses and ustiviruses are concerned, no direct correlation between viral infection and symptom was ever evidenced to our knowledge. Actually, many of the amalgaviruses have been discovered randomly through transcriptomic data [29,48,49,50], probably illustrating a persistent nature. It seems that those viral species do not represent relevant candidates for biocontrol purpose given the absence of an impact on the host, even though phenotype alternation under particular conditions cannot be ruled out.

Eventually, we performed a screening of other *F. culmorum* and we gave evidences for the presence of the virus in two other strains from Europe. In the future, it will be interesting to obtain the sequences of those potential FcV1 isolates as well as to test more strains in order to shed light on the nucleotide diversity of this virus.

In summary, in this study we have described FcV1, a novel monopartite mycovirus infecting *F. culmorum*. To our knowledge, it is the first full genomic sequence for a virus infecting this fungal species. Our studies also broaden the diversity of mycovirus belonging to “Unirnaviridae” and supports the proposition of this taxon being considered as a new viral family.

## Figures and Tables

**Table 1:** Genomic features of unirnaviruses (updated and adapted from Depierrieux et al. [8]).

Virus Name	Abbreviation	Accession Number	Genome Size (bp)	5′UTR (bp)	3′UTR (bp)	ORF1p (aa)	ORF1+2p (aa)	Reference
Penicillium janczewskii Beauveria bassiana-like virus 1	PjBlV1	KT601106	2890 ^1^	10	110	314	-^1^	[29]
Beauveria bassiana non-segmented virus 1	BbNV1	LN610699	3218	320	79	315	926	[30]
Alternaria longipes dsRNA virus 1	AlRV1	KJ817371	3415	318	107	394	997	[31]
Ustilaginoidea virens unassigned RNA virus HNND 1	UvURVHNND1	KR106133	2903	33	72	314	932	[32]
Ustilaginoidea virens RNA virus M	UvRVM	KJ101567	2714 ^1^	-^1^	-^1^	-^1^	-^1^	[33]
Colletotrichum higginsianum non-segmented dsRNA virus 1	ChNRV1	KM923925	2923	16	106	324	933	[27]
Trichoderma harzianum mycovirus 1	ThV1	MH155602	3160	113	111	379	979	[34]
Combu-like dsRNA virus 1	ClV1	MH990637	2992	12	188	326	930	Unpublished
Penicillium miczynskii RNA virus 1	PmRV1	MK584820	2891	226	134	238	-^2^	[35]
Fusarium culmorum virus 1	FcV1	MN187541	2898	14	106	314	926	This study

^1^ Apparently not fully sequenced. ^2^ No -1 PRF motif found.

## Supplementary Materials

The following are available online at https://www.mdpi.com/1999-4915/12/5/523/s1, Figure S1: Treatment of the dsRNA with DNase I and S1 nuclease. Figure S2: Amplicons obtained after RT-PCR on the dsRNA. Figure S3: CC prediction for proteins obtained following HMMERsearch on unirnaviruses ORF1p. Figure S4: Phylogenetic tree of FcV1 ORF2 and related RdRp using Bayesian method. Figure S4: Analysis of FcV1 in other *F. culmorum* strains. Table S1: Accession numbers of ORF1p of unirnaviruses, amalgaviruses and ustiviruses, Table S2: RdRp-domain containing ORF. Table S3: Description of *F. culmorum* strains screened for the presence of FcV1.
