# Supplementary material for: Description of a Novel Mycovirus in the Phytopathogen Fusarium culmorum and a Related EVE in the Yeast Lipomyces starkeyi
Source: Viruses. 2020 May 9;12(5):523. doi: 10.3390/v12050523 (PMC7290986; doi:10.3390/v12050523)
Supplement: Supplementary file 1 [file viruses-12-00523-s001.pdf]

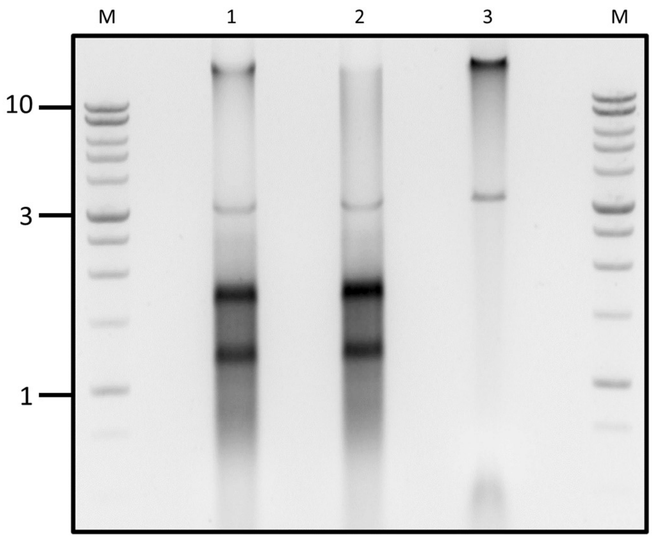

Figure S1. Electrophoresis agarose (1%) gel showing sample obtained after dsRNA extraction from *F. culmorum* A104-1. M: DNA weight markers (Promega 1 kb ladder). Lane 1: non-treated extracted sample. Lane 2: sample treated with DNase I. Lane 3: sample treated with S1 nuclease. Numbers on the left refer to chosen DNA molecular weights expressed in kilobase.

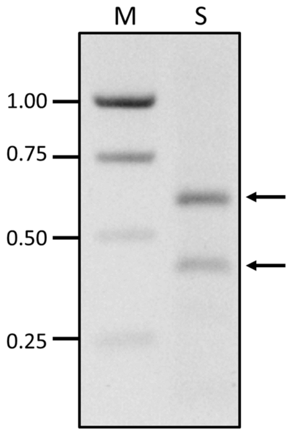

Figure S2. Electrophoresis agarose (1%) gel showing the two amplicons obtained (black arrows) after RT-PCR on the dsRNA using the degenerate primer. M: DNA weight markers (Promega 1 kb ladder). S: sample. Numbers on the left refer to chosen DNA molecular weights expressed in kilobase.

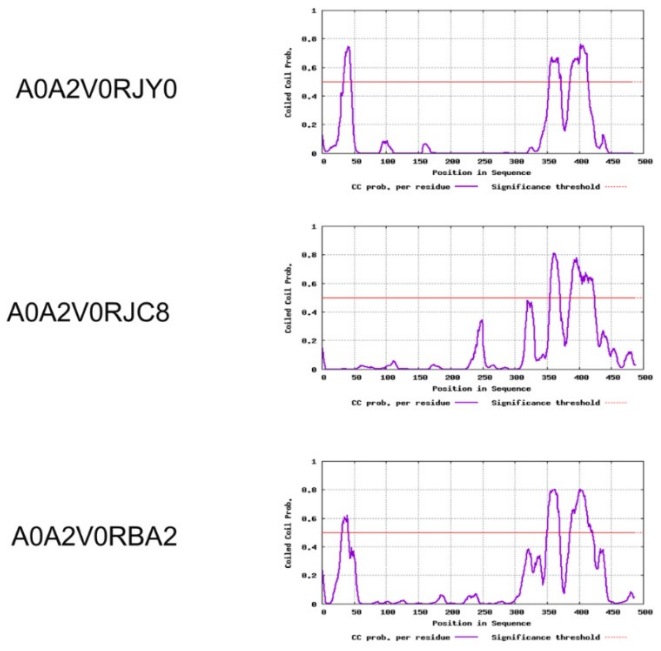

Figure S3. Coiled-coil prediction using Deepcoil for the three proteins obtained after HMMERsearch on unirnaviruses ORF1p.

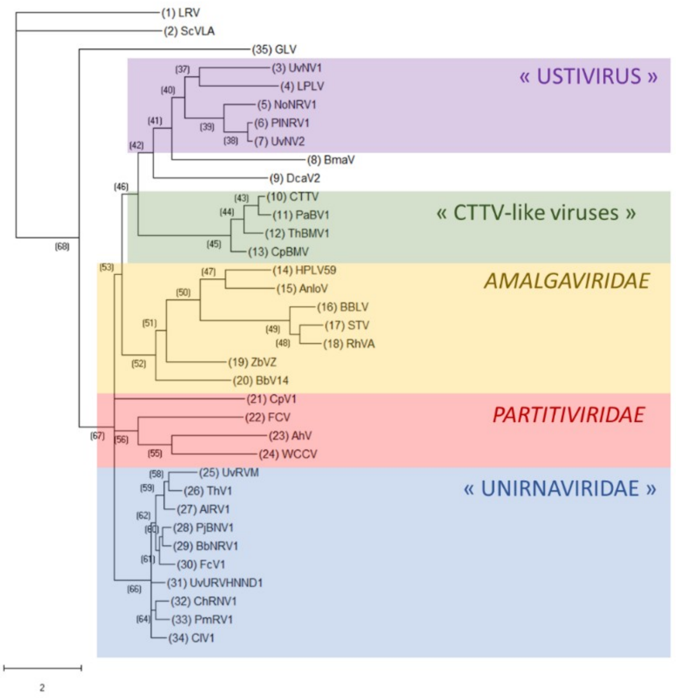

Figure S4. Phylogenetic tree of FcV1 ORF2 and related RdRp. The numbers next to nodes refer the support values. Members of the family *Totiviridae* (GLV, LRV and ScVLA) were taken to root the initial tree.

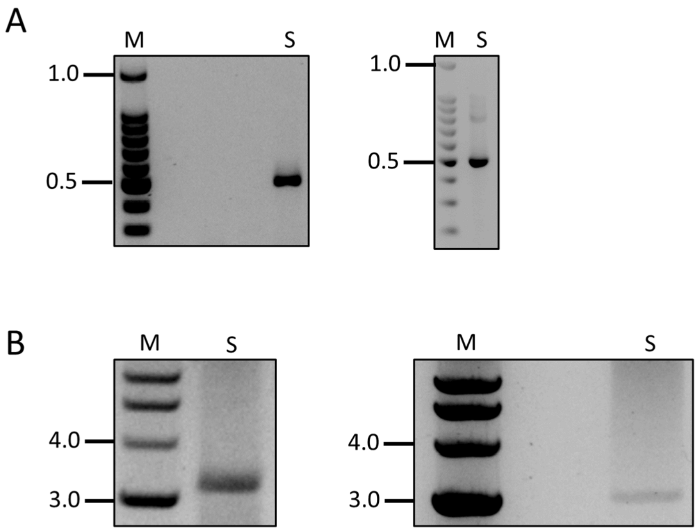

Figure S5. Analysis for the presence of FcV1 in other *F. culmorum* strains. (a) Electrophoresis agarose (1%) gels showing amplicon obtained after RT-PCR on RNA from the strain Fc30 (left) and PVS-Fu353 (right). M: Promega 100 bp. S: sample; (b) Electrophoresis agarose (1%) gels showing dsRNA extracted from the strain Fc30 (left) and PVS-Fu353 (right). M: Promega 1 kb. S: sample. For all gels, numbers on the left refer to chosen DNA molecular weights expressed in kilobase.

Table S1. Accession numbers of ORF1p of unirnaviruses, amalgaviruses and ustiviruses.

	**Accession number of ORF1p**
**"UNIRNAVIRIDAE"**	
Penicillium janczewskii Beauveria bassiana-like virus 1 (PjBlV1)	ALO50134.1
Beauveria bassiana non-segmented virus 1 (BbNV1)	CEF90231.1
Alternaria longipes dsRNA virus 1 (AlRV1)	YP_009052468.1
Ustilaginoidea virens unassigned RNA virus HNND 1 (UvURVHNND1)	YP_009154708.1
Colletotrichum higginsianum non-segmented dsRNA virus 1 (ChNRV1)	YP_009177216.1
Trichoderma harzianum mycovirus 1 (ThV1)	AYU71188.1
Combu-like dsRNA virus 1 (ClV1)	QAB47443.1
Penicillium miczynskii RNA virus 1 (PmRV1)	QDB74979.1
Fusarium culmorum virus 1 (FcV1)	This study
**"USTIVIRUS"**	
Purpureocillium lilacinum nonsegmented virus 1 (PlNV1)	AOO52901.1
Ustilaginoidea virens nonsegmented virus 1 (UvNV-1)	AIE77247.1
Nigrospora oryzae nonsegmented RNA virus 1 (NoNRV1)	ALR87112.1
Ustilaginoidea virens nonsegmented virus 2 (UvNV-2)	YP_009553681.1
AMALGAVIRUS	
Blueberry latent virus (BBLV)	BBI01013.1
Rhododendron virus A (RhVA)	YP_003868437.1
Southern tomato virus (STV)	QDF44095.1
Vicia cryptic virus (VCV)	YP_272125.1
Allium cepa amalgavirus 1 (AcAV1)	YP_009447920.1
Anthoxanthum odoratum amalgavirus 1 (AoAV1)	YP_009551563.1
Camellia oleifera amalgavirus 1 (CoAV1)	YP_009551565.1
Phalaenopsis equestris amalgavirus 1 (PeAV1)	YP_009552084.1

Tables S2. RdRp-domain containing ORF.

	**Accession number of RdRp-ORF**
**"UNIRNAVIRIDAE"**	
Penicillium janczewskii Beauveria bassiana-like virus 1 (PjBlV1)	ALO50135.1
Beauveria bassiana non-segmented virus 1 (BbNV1)	YP_009154711.1
Alternaria longipes dsRNA virus 1 (AlRV1)	YP_009052469.1
Ustilaginoidea virens unassigned RNA virus HNND 1 (UvURVHNND1)	YP_009154709.1
Colletotrichum higginsianum non-segmented dsRNA virus 1 (ChNRV1)	AIW81425.1
Trichoderma harzianum mycovirus 1 (ThV1)	AYU71187.1
Combu-like dsRNA virus 1 (ClV1)	QAB47444.1
Penicillium miczynskii RNA virus 1 (PmRV1)	QDB74980.1
Ustilaginoidea virens RNA virus M (UvRVM)	YP_009094186.1
Fusarium culmorum virus 1 (FcV1)	This study
**"USTIVIRUS"**	
Purpureocillium lilacinum nonsegmented virus 1 (PlNV1)	AOO52902.1
Ustilaginoidea virens nonsegmented virus 1 (UvNV-1)	AIE77248.1
Nigrospora oryzae nonsegmented RNA virus 1 (NoNRV1)	ALR87111.1
Ustilaginoidea virens nonsegmented virus 2 (UvNV-2)	AXR76113.1
**"AMALGAVIRIDAE"**	
Blueberry latent virus (BBLV)	Retrieved from NC_014593.1
Rhododendron virus A (RhVA)	Retrieved from NC_014481.1
Southern tomato virus (STV)	Retrieved from MG808383.1
Zygosaccharomyces bailii virus Z (ZbVZ)	NP_624325.1
Antonospora locustae virus 1 (AnloV)	Retrieved from KX525322.1
Beihai barnacle virus 14 (BbV14)	APG78182.1
Hubei partiti-like virus 59 (HPLV59)	APG78262.1
**CTTV-like viruses**	
Curvularia thermal tolerance virus (CTTV)	ALO61394.1
Cryphonectria parasitica bipartite mycovirus 1 (CpBMV)	YP_007985675.1
Penicillium aurantiogriseum bipartite virus 1 (PaBV1)	YP_009182335.1
Trichoderma harzianum bipartite mycovirus 1	YP_009553330.1
**PARTITIVIRIDAE**	
Fig cryptic virus (FCV)	YP_004429258.1
Cryptosporidium parvum virus 1 (CpV1)	AAC47805.1
Atkinsonella hypoxylon virus (AhV)	NP_604475.1
White clover cryptic virus 1 (WCCV)	YP_086754.1
**UNCLASSIFIED**	
Bryopsis mitochondria-associated virus (BmaV)	BAA25883.1
Diatom colony-associated dsRNA virus 2 (DcaV2)	YP_009551448.1
**TOTIVIRIDAE**	
Giardia lamblia virus (GLV)	Retrieved from NC_003555.1
Leishmania RNA virus (LRV)	Retrieved from NC_002064.1
Saccharomyces cerevisiae virus L-A (ScVLA)	NP_620495.1

Table S3. Description of *F. culmorum* strains screened for the presence of FcV1.

	Year	Country	Place	Plant	Tissue	Code
1	2010	Italy	Pozzo S. Nicola (Sassari)	Wheat	Ear	PVS-Fu 353
2	2009	Italy	Ottava (Sassari)	Wheat	Ear	PVS-Fu 368
3	2009	Italy	Ottava (Sassari)	Wheat	Ear	PVS-Fu 373
4	2001	Italy	Ussana (Cagliari)	Wheat	Crown	PVS-Fu 410
5	1972	Belgium	Nil-Saint-Vincent	Barley	Grain	MUCL 18524
6	1980	Finland	-	Barley	Grain	MUCL 28166
7	1980	Finland	-	Barley	Grain	MUCL 28167
8	2002	France	-	Wheat	Grain	MUCL 43772
9	1927	Canada	-	Avoine	-	MUCL 43791
10	1962	Belgium	Heverlee	*A.officinalis*	Stem	MUCL 43792
11	1951	Netherland	Lisse	Hyacinthus	Root	MUCL 43793
12	2002	Belgium	-	Wheat	Grain	MUCL 43794
13	2002	Belgium	-	Wheat	Grain	MUCL 43795
14	2002	Belgium	-	Wheat	Grain	MUCL 43796
15	2002	Belgium	-	Wheat	Grain	MUCL 43797
16	2002	Belgium	-	Wheat	Grain	MUCL 43798
17	2011	Belgium	Koksijde	-	-	861
18	2011	Belgium	Verrebroek	Wheat	-	2723
19	2014	Italy	Agugliano (Ancona)	Durum wheat	Grain	Culm1
20	2014	Italy	Agugliano (Ancona)	Durum wheat	Grain	Culm2
21	2014	Italy	Agugliano (Ancona)	Durum wheat	Grain	Culm3
22	2014	Italy	TorMancina (Rome)	Durum wheat	Crown	ER 1998
23	1994	Russia	St. Petersburg	Winter wheat	-	Russ 1.1.1
24	1994	Russia	St. Petersburg	Winter wheat	-	Russ 4.1.1
25	1994	Russia	St. Petersburg	Winter wheat	-	Russ 7.1.1
26	1994	Russia	St. Petersburg	Winter wheat	-	Russ 23.1.1
27	2003	Serbia	Sremska Mitrovica	Wheat	Grain	MRIZP 209
28	2003	Serbia	Sremska Mitrovica	Wheat	Grain	MRIZP 210
29	1997	Serbia	Badince	Maize	Root	MRIZP 1239
30	1997	Serbia	Badince	Maize	Root	MRIZP 1318
31	2009	Serbia	Zemun Polje	Barley	Grain	MRIZP 2073
32	2003	Serbia	Sremska Mitrovica	Wheat	Grain	MRIZP 2830
33	2012	Serbia	Sremska Mitrovica	Weed	Grain	MRIZP 3762
34		Turquey	Eskisehir, central anatolia	*A. cylindrica*	Root	En23-2
35	1984	Germany	Wetze	Winter wheat		FC 8
36	1984	Germany	Grünbach	Winter wheat		FC 10
37	1987	Germany	Söllingen	Winter wheat		FC 15
38	1991	Germany	Vaihingen	Emmer		FC 30
39	1993	Portugal	Luz	Winter durum		FC 90
40	1995	Portugal	Luz	Wheat		FC 97
41	1987	Netherland	Wageningen	-		FC 46
42	1952	Netherland	-	Wheat	Grain	FC 60
43	1997	UK	Louth	Wheat		F98/11
44	1998	UK	Barhill, Cambridge	Wheat		F97/7
45	1998	UK	Hertfordshire	Wheat		UK99
46	1984	Poland	Swadzim	Maize		KF 194
47	1986	Poland	Radzików	Wheat		KF 846
48	1986	Poland	Radzików	Maize		KF 915
49	-	NewZealand	-	Wheat		KF 2102
50	-	UK	-	Wheat		KF 2104
51	2009	Poland	-	Wheat		KF 3852
52	2010	Turquey	Eskisehir, central anatolia	*T. aestivum*	Root	H010W21-2K
53	2014	Turquey	Tokat, northern anatolia	*T.aestivum*	Grain	H014W9-2B
